# A Well Merited Appointment

**Published:** 1897-09

**Authors:** 


### A WELL-MERITED APPOINTMENT.

Readers of the Journal will be pleased to learn that Dr. S. D. Brimhall, a graduate of the University of Pennsylvania, has recently been selected for field veterinarian by the Minnesota State Board of Health to assist Dr. Reynolds, and is already doing most excellent work for that State. Salary, $1800. The doctor is very kindly remembered in Philadelphia by the veterinarians who were in college with him, and by the veterinary faculty of the University as a conscientious student and a thorough gentleman.

The Minnesota State Board of Health and the State Experiment Station are now co-operating in field experiments with hog-cholera-serum. Dr. Peters is doing the laboratory work and Dr. Reynolds is planning and managing the field-work. Dr. Brimhall is now in the southern part of the State engaged in this work.

